# Supplementary material for: DRG2 Deficient Mice Exhibit Impaired Motor Behaviors with Reduced Striatal Dopamine Release
Source: Int J Mol Sci. 2019 Dec 20;21(1):60. doi: 10.3390/ijms21010060 (PMC6981536; doi:10.3390/ijms21010060)
Supplement: Supplementary file 1 [file ijms-21-00060-s001.zip › ijms-652804/Supplemental Fig legends and M&M.docx]

**Supplemental figure legends**

**Supplemental Fig 1. Expression pattern of DRG2 in mouse brain and effect of DRG2 expression on the brain weight**. (**A and B)** Immunohistochemical localization of DRG2 in (**A**) hippocampus (HIP) and (**B**) cerebral cortex (CTX). Coronal sections of mouse brain were incubated with antibodies against DRG2, neuronal marker Tuj1, astrocytes marker GFAP, and microglia marker Iba1. Nuclei were stained with DAPI. Scale bar 20 μm. (**C**) Western blot analysis for DRG2 in neurons, astrocytes, and microglia isolated from mouse embryo brain as described in “Materials and Methods”. The same amount of total protein (10 μg total protein per each sample) was loaded. (**D**) Comparision of brains of 3-month old DRG2^+/+^, DRG2^+/-^, and DRG2^-/-^ mice. Right, representative images of brains of DRG2^+/+^, DRG2^+/-^, and DRG2^-/-^ mice. Data represent brain weight (means ±SEM, n=6 per each group). (**E**) DAPI staining of coronal sections of 3-month old DRG2^+/+^ and DRG2^-/-^ mice. Top two panels, images of whole coronal sections. Scale bars, 1 mm. Bottom two panels, the boxed regions in “Top two panels” were viewed at higher magnification. Scale bars, 500 μm.

**Supplemental Fig 2. DRG2 deficient does not alter the population of TH neurons**. Immunohistochemical staining of DRG2^+/+^ and DRG2^-/-^ mice brains for TH neurons. Serial coronal sections of 24-month old DRG2^+/+^ and DRG2^-/-^ mice brains were incubated with antibody against TH. TH neurons in (**A**) striatum (Str), (**B**) substantia nigra (SN), and VTA were visualized by incubating with streptavidin–horseradish peroxidase complex. Scale bars, 500 μm. (**C**) TH neurons in SN, Str, Nac core, and Nac shell were visualized by incubating with Alexa-488-labeled secondary antibody. Scale bars, 100 μm. Data represent TH intensity in each brain regions. (means ± SEM, n=3 per each group).

**Supplemental Materials and Methods**

***Isolation of neurons, astrocytes, and microglia***

Primary cortical neuron cultures were prepared from 16-day-old embryonic mice as described previously [1, 2]. Briefly, mouse embryos were decapitated, and the brains were rapidly removed and placed in a culture dish containing HBSS (Thermo Scientific, Waltham, MA). Cortices were isolated, transferred to a conical tube and washed twice in HBSS (Thermo Scientific). Cortical tissues were enzymatically digested by prewarmed papain (20 units/ml) (Worthington Biochemical Corporation) and DNase I (0.005%) for 30 min at 37 °C. The tissues were mechanically dissociated (triturated) with 1000 μL and 200 μL pipette tips for complete tissue homogenization. The cortical cells were centrifuged at 800 rpm for 10 min at room temperature, and the dissociated cells obtained were seeded onto plates coated with poly-D-lysine (Sigma-Aldrich) in neurobasal media containing 2 mM glutamine (Thermo Scientific), N2 supplement (Thermo Scientific), B27 supplement (Thermo Scientific), and penicillin-streptomycin (Thermo Scientific). The culture media were changed initially after 5 days and every 3 days thereafter, and cells were used after being cultured for 18 days.

Astrocyte and microglia cultures were prepared from the brains by the method of McCarthy and de Vellis [3]. Briefly, whole brains were homogenized in a 70-μm strainer. Cells were seeded in T75 culture flasks or 100mm culture dish. Cells were grown at 37 °C in a humidified atmosphere containing 5% CO_2_. Culture media were changed initially after 5 days and every 2 days thereafter, and cells were used after culture for 14-21 days. Secondary pure astrocyte cultures were obtained by shaking mixed glial cultures at 250 rpm for 4 hr, and then, the culture medium was discarded. Astrocytes were dissociated using trypsin-EDTA (Life Technologies) and then centrifuged at 2000 rpm for 30 min. The astrocytes obtained were seeded onto plates in DMEM (Life Technologies) supplemented with 10% heat-inactivated fetal bovine serum (FBS; Gibco) and penicillin-streptomycin (Thermo Scientific). Pure microglia cultures were performed using the mild trypsinization solution (155 mM NaCl, 2.9 mM Na_2_HPO_4_-7H_2_O, 1 mM KH_2_PO_4_, 0.2 mM EDTA, 0.5 mM CaCl_2_, 0.05% Trypsin-EDTA (Ph 7.4)) Briefly, 5ml of mild trypsinization solution was added into each 100mm dish and the dish was incubated at 37 °C for 30-60 min. The detached layer of astrocytes was discarded and microglia were dissociated using trypsin-EDTA (Life Technologies), and then centrifuged at 2000 rpm for 30 min. The microglia obtained were seeded onto plates in DMEM (Life Technologies) supplemented with 10% heat-inactivated FBS(Thermo Scientific) and penicillin-streptomycin (Thermo Scientific).

**Supplemental references**

1. Araki, W., et al., *Overexpression of presenilin-2 enhances apoptotic death of cultured cortical neurons.* Ann N Y Acad Sci, 2000. **920**: p. 241-4.

2. Enokido, Y., et al., *Basic fibroblast growth factor rescues CNS neurons from cell death caused by high oxygen atmosphere in culture.* Brain Res, 1992. **599**(2): p. 261-71.

3. McCarthy, K.D. and J. de Vellis, *Preparation of separate astroglial and oligodendroglial cell cultures from rat cerebral tissue.* J Cell Biol, 1980. **85**(3): p. 890-902.
