# Supplementary figures and images for: DRG2 Deficient Mice Exhibit Impaired Motor Behaviors with Reduced Striatal Dopamine Release
Source: Int J Mol Sci. 2019 Dec 20;21(1):60. doi: 10.3390/ijms21010060 (PMC6981536; doi:10.3390/ijms21010060)

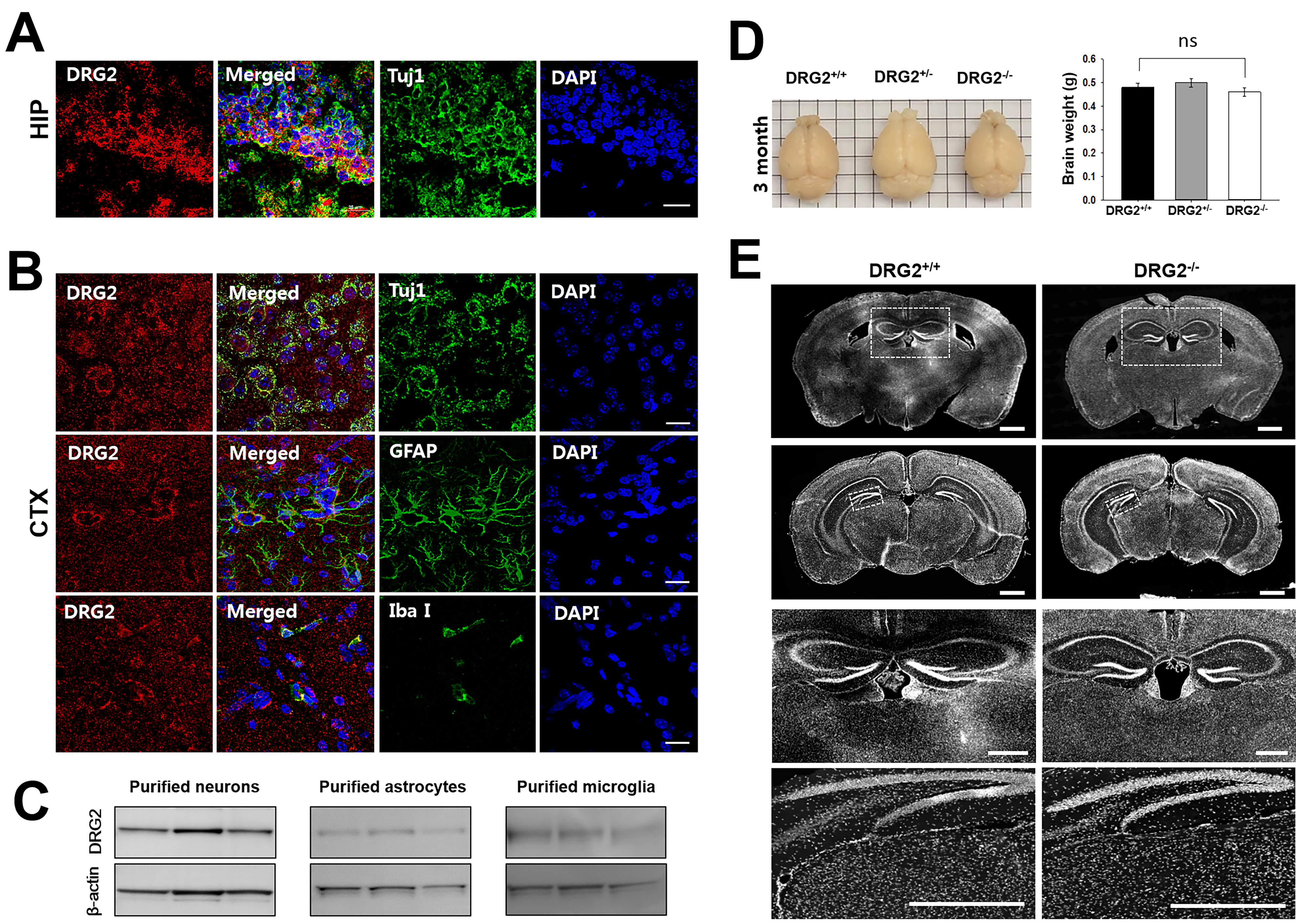

Supplement: Supplementary file 1 [file ijms-21-00060-s001.zip › ijms-652804/Figure S1.tif]

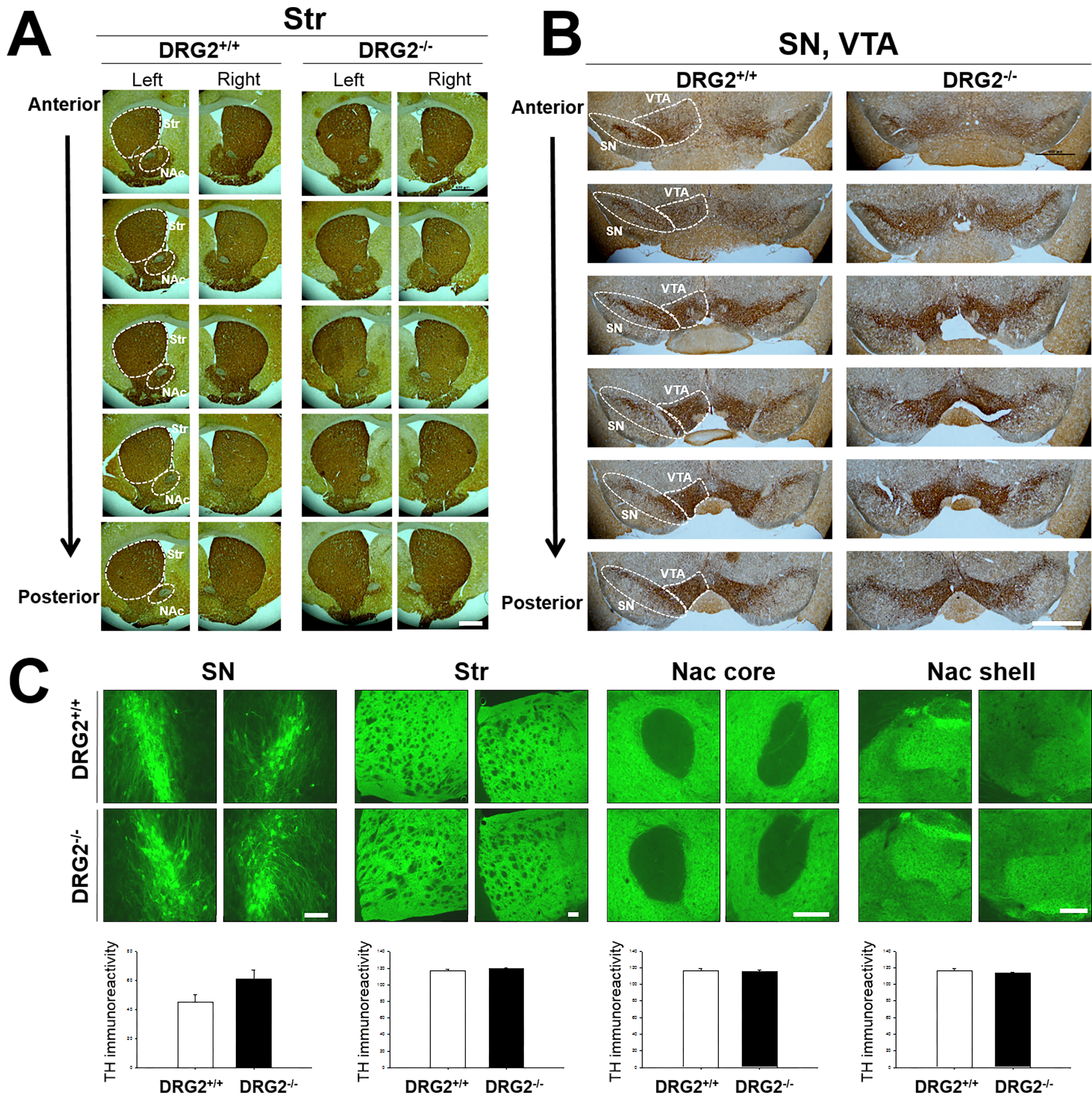

Supplement: Supplementary file 1 [file ijms-21-00060-s001.zip › ijms-652804/Figure S2.tif]
